# Evaluation of Platelet Indices in Coronary Artery Disease: An Institutional Study

**DOI:** 10.7759/cureus.82130

**Published:** 2025-04-12

**Authors:** Kamala Kannappalli, Kalyani Raju, Subhashish Das, Prabhakar K, Snigdha Sinha

**Affiliations:** 1 Pathology, Sri Devaraj Urs Medical College, Kolar, IND; 2 General Medicine, Sri Devaraj Urs Medical College, Kolar, IND

**Keywords:** coronary artery disease, grace score, mpv, pdw, platelet indices

## Abstract

Introduction

Coronary artery disease (CAD) is the most prevalent form of heart disease and continues to be a major cause of death for both men and women. In CAD, platelet size tends to increase due to the essential role platelets play in hemostasis. Exploring the relationship between platelet and WBC indices may improve screening strategies for identifying high-risk individuals.

Aim

This study aims to evaluate platelet indices - specifically mean platelet volume (MPV), platelet large cell ratio (PLCR), and platelet distribution width (PDW) - in patients with CAD. It also seeks to compare these indices between patients with myocardial infarction and healthy individuals while identifying risk factors that may contribute to the development of CAD.

Materials and methods

A prospective study was carried out over a period of six months, involving 100 patients diagnosed with CAD. Platelet indices, including MPV, PLCR, PDW, and platelet-to-lymphocyte ratio (PLR), were measured using the Sysmex XN 1000 M2 automated hematology analyzer (Sysmex Corporation, Kobe, Japan).

Results

When comparing the CAD group to the control group, there was no significant difference in platelet count. However, PLR, MPV, PDW, PLCR, and the Global Registry of Acute Coronary Events score showed highly significant differences between the two groups, suggesting their potential relevance in disease assessment.

Conclusions

Readily available diagnostic markers such as PLR, MPV, and PDW, along with other platelet indices, may serve as useful indicators for assessing the risk of CAD. These findings highlight the potential of combining such markers with conventional cardiac biomarkers like troponin, CRP, and creatine kinase-MB to aid in the early detection and prediction of CAD.

## Introduction

In developing countries, coronary artery disease (CAD) is the most common type of heart disease and remains a leading cause of death among both males and females. It occurs when the arteries harden and narrow due to the accumulation of cholesterol, eventually forming a plaque known as atherosclerosis. CAD contributes significantly to global health burdens, accounting for approximately 12 million deaths annually in developing nations [1].

Platelets play a critical role in cardiovascular disease, and their size is a key indicator of platelet activity. In CAD, platelet size tends to increase due to their involvement in hemostasis and repair mechanisms associated with atherothrombosis [2]. Larger platelets - typically around 7 µm in size - are more reactive and release greater amounts of proinflammatory and prothrombotic mediators, which contribute to coronary events. These platelets are also more metabolically and enzymatically active compared to smaller ones, which range from 1.5 to 3 µm in size [3-6]. In myocardial infarction, platelet parameters such as mean platelet volume (MPV) and platelet large cell ratio (PLCR) often show abnormalities even before traditional biochemical markers become evident [1].

In addition to platelet parameters, WBC indices are also recognized as potential predictors of adverse cardiovascular outcomes. Platelet activity can be measured using MPV through hematology analyzers. Recent research on CAD has identified the platelet-to-lymphocyte ratio (PLR) as a novel prognostic marker [6-8]. However, there is a limited number of studies investigating the association between these hematological parameters and cardiovascular events.

The Global Registry of Acute Coronary Events (GRACE) score is a well-established tool used to estimate the risk of mortality in patients with acute coronary syndrome (ACS) [5,6,9]. It incorporates eight clinical and laboratory parameters: age, cardiac arrest at admission, Killip class, ST-segment deviation, serum creatinine levels, elevated cardiac enzymes, heart rate, and systolic blood pressure. The GRACE score ranges from 2 to 372, classifying patients into three risk categories: low (1-108), intermediate (109-140), and high (>140) [6].

This study aims to evaluate platelet indices - MPV, PLCR, and platelet distribution width (PDW)-as well as the PLR and GRACE risk scores in patients diagnosed with CAD. It also seeks to compare these platelet indices between patients with myocardial infarction and healthy controls and to identify potential risk factors associated with the development of CAD.

## Materials and methods

The present study was designed as a descriptive cross-sectional study and was conducted at Sri Devaraj Urs Medical College, RL Jalappa Hospital and Research Centre, Kolar, Karnataka, over a period of six months, from August 2023 to January 2024.

Sample size and study subjects

The study included 100 patients who were provisionally diagnosed with CAD and whose diagnoses were confirmed through ECG, angiography, or cardiac markers. Additionally, 100 age- and sex-matched healthy individuals were selected as controls.

Inclusion criteria

A total of 100 patients diagnosed with CAD were included in the study. The control group comprised 100 healthy individuals who presented for routine health checkups and had no history of comorbidities such as coronary heart disease, myocardial infarction, or renal failure. Only patients with a provisional diagnosis of CAD, later confirmed by ECG, angiography, or cardiac markers, were included.

Exclusion criteria

Patients presenting with non-cardiac chest discomfort, chronic CAD, recurrent myocardial infarction, a history of renal disease, previous coronary interventions or coronary artery bypass grafting, inflammatory rheumatic disease, chronic obstructive pulmonary disease, or those receiving oral anticoagulants were excluded. Additionally, individuals who did not provide informed consent were not included in the study.

Method of collection of data

Venous blood samples were collected in vacutainers containing di-potassium EDTA and processed within one hour of collection. Platelet indices - platelet count, MPV, PLCR, and PDW - as well as the PLR, were measured in both cases and controls using the Sysmex XN-1000 M2 automated hematology analyzer (Sysmex Corporation, Kobe, Japan). Serum creatinine and cardiac markers, including troponin T and creatine kinase-MB, were analyzed using a fully automated Siemens biochemistry analyzer (Siemens Healthineers, Forchheim, Germany). D-dimer levels were measured using the SAGO STA Compact fully automated coagulation analyzer.

The study received approval from the Institutional Ethics Committee and was conducted in accordance with the ethical standards of the 1975 Declaration of Helsinki and its subsequent amendments. Written informed consent was obtained from all participants prior to their inclusion.

Statistical analysis

Data was recorded using Microsoft Excel (Microsoft Corporation, Redmond, WA, USA) and analyzed with IBM SPSS Statistics for Windows, Version 22.0 (Released 2013; IBM Corp., Armonk, NY, USA). Categorical data was expressed as frequencies and proportions, and the chi-square test or Fisher’s exact test (for 2 × 2 tables) was used to assess the significance of qualitative variables. Continuous variables were presented as mean and SD, and the independent t-test was used to evaluate differences in means between groups.

Receiver operating characteristic (ROC) curves were generated to assess the diagnostic value of various parameters in relation to CAD. Optimal cutoff points were identified to calculate sensitivity, specificity, and positive and negative predictive values. An area under the ROC curve (AUC) of 0.5 indicated no diagnostic ability, while an AUC greater than 0.8 was considered indicative of good predictive value.

Graphs and visual data representations were created using Microsoft Excel and Word (Microsoft Corporation). A p-value of less than 0.05 was considered statistically significant, assuming all standard conditions of statistical testing were met.

## Results

The characteristics of the CAD patients and controls are summarized in Table 1. The mean age of the CAD patients was 58.76 ± 15.10 years, while the mean age of the controls was 56.56 ± 16.90 years, with no statistically significant difference between the two groups (p = 0.333). There was no significant difference in platelet and lymphocyte counts between the CAD patients and controls. However, the PLR, MPV, PDW, PLCR, and GRACE scores were significantly higher in CAD patients compared to the control group, with p-values of <0.013, 0.001, 0.002, 0.001, and 0.001, respectively.

**Table 1:** Comparison of various parameters between the case and control groups The asterisks indicate statistical significance: p < 0.05 is considered statistically significant (*), and p < 0.01 is considered highly statistically significant (**). GRACE, Global Registry of Acute Coronary Events; MPV, mean platelet volume; PDW, platelet distribution width; PLR, platelet-to-lymphocyte ratio

Parameters	Cases		Control		p-Value
	Mean	SD	Mean	SD	
Age (years)	58.76	15.1	56.56	16.9	0.333
Platelet count (per cumm)	284,680	107,633	263,690	94,030.6	0.144
Lymphocyte count (%)	1.84	1.83	1.46	1.09	0.077
PLR	293.86	224.21	220.41	188.38	0.013*
MPV (femtoliter)	10.75	1.98	9.97	1	0.001**
PDW (%)	11.01	2.42	9.74	3.14	0.002*
PLCR (femtoliter)	29.5	9.64	24.14	8.29	<0.001**
GRACE score	141.24	38.8	86.42	30.53	<0.001**

**Figure 1:**
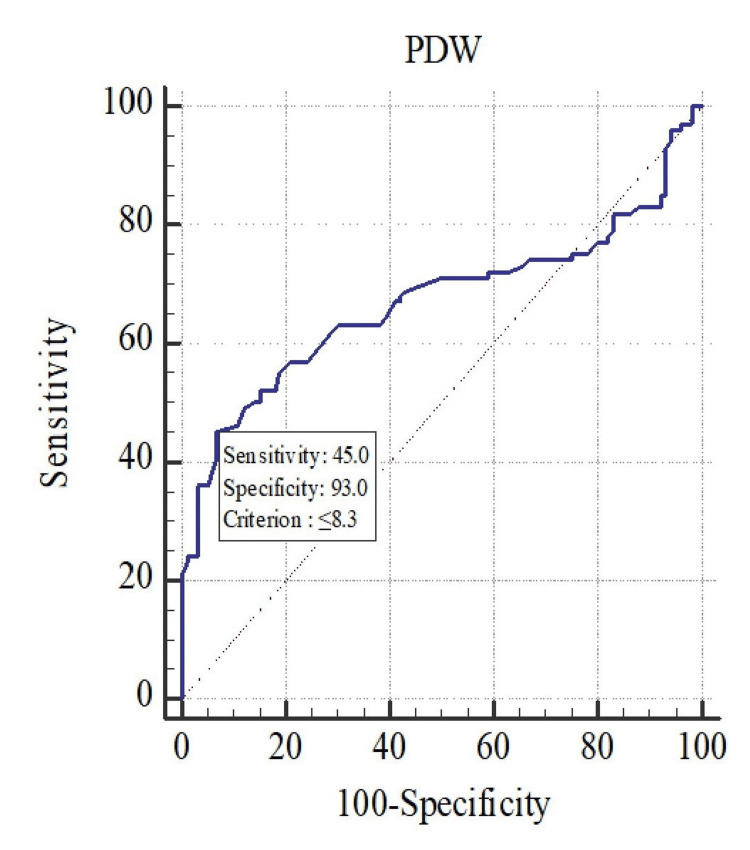
ROC curve analysis for the prediction of CAD using PDW CAD, coronary artery disease; PDW, platelet distribution width; ROC, receiver operating characteristic

**Figure 2:**
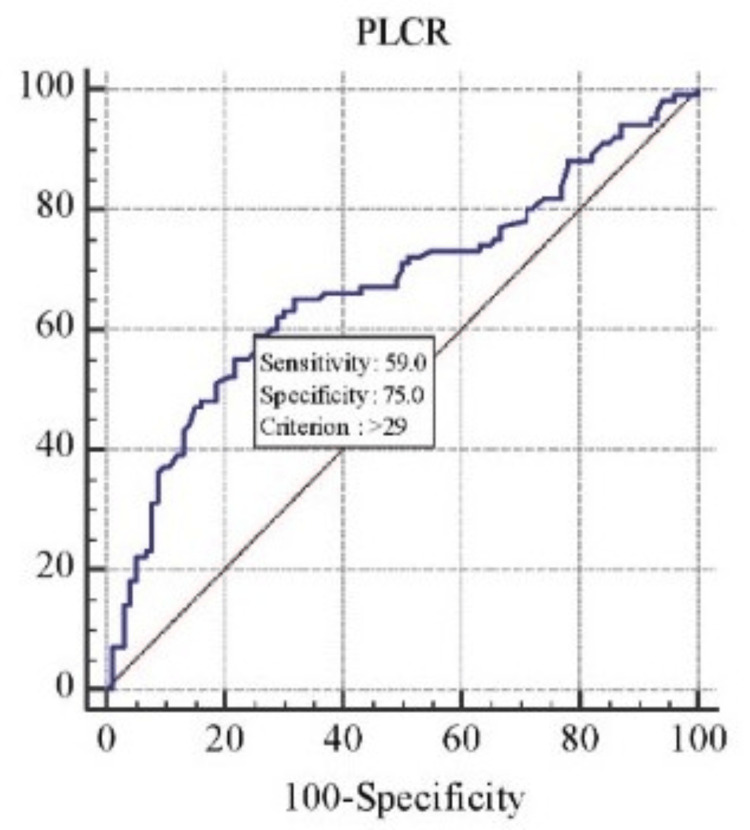
ROC curve analysis for the prediction of CAD using PLCR CAD, coronary artery disease; PLCR, platelet large cell ratio; ROC, receiver operating characteristic

**Figure 3:**
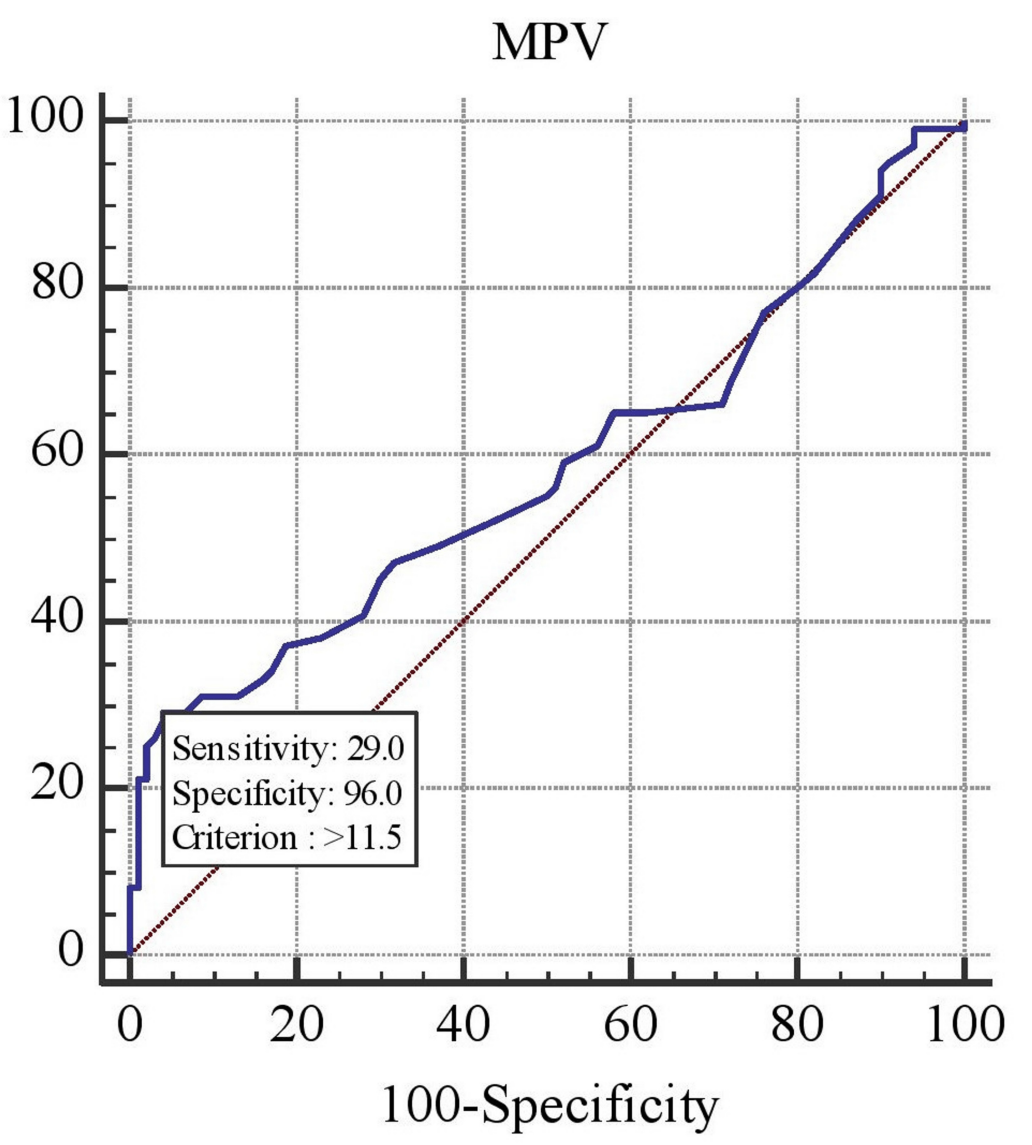
ROC curve analysis for the prediction of CAD using MPV CAD, coronary artery disease; MPV, mean platelet volume; ROC, receiver operating characteristic

Coronary angiography was employed in the diagnosis of coronary atherosclerosis and CAD, with the diagnostic model evaluated using ROC curve analysis. The accuracy of platelet indices in predicting CAD was assessed through ROC curve analysis. In logistic regression, ROC curves are used to determine the optimal cutoff value for predicting whether a new observation is classified as a “failure” (0) or “success” (1). The ROC curves were generated using data from all patients, as shown in Figure 1, Figure 2, and Figure 3, and the analysis is summarized in Table 2.

**Table 2:** ROC curve analysis for prediction of CAD by various parameters AUC, area under the ROC curve; CAD, coronary artery disease; MPV, mean platelet volume; PDW, platelet distribution width; PLCR, platelet large cell ratio; ROC, receiver operating characteristic

Parameters	AUC	SE	95% CI	p-Value
PDW (%)	0.67	0.04	0.600-0.735	<0.001
PLCR (femtoliters)	0.67	0.03	0.600-0.735	<0.001
MPV (femtoliters)	0.583	0.0409	0.511-0.652	0.04

The AUC methodology was employed to assess the discrimination ability of various parameters. ROC analysis of PDW demonstrated a strong correlation with CAD, yielding an AUC of 0.670 (p < 0.001, Figure 2). ROC analysis was also used to determine the cutoff value for PLCR, which showed a sensitivity of 59% and specificity of 75% (AUC = 0.670, 95% CI: 0.6-0.735, p < 0.001). A PDW cutoff value of 8.3% exhibited a sensitivity of 45% and specificity of 93% for predicting CAD. Additionally, a higher MPV cutoff of 11.5 fL showed 29% sensitivity and 96% specificity for predicting CAD. The cutoff value for PLCR in predicting CAD was greater than 2, with 75% specificity and 59% sensitivity, as shown in Table 3.

**Table 3:** Cutoff values for various parameters with sensitivity, specificity, PPV, and NPV for the prediction of CAD CAD, coronary artery disease; MPV, mean platelet volume; NPV, negative predictive value; PDW, platelet distribution width; PLCR, platelet large cell ratio; PPV, positive predictive value

Parameters	Cutoff	Sensitivity	Specificity	PPV	NPV
PDW (%)	≤8.3	45	93	86.5	62.8
PLCR (femtoliters)	>29	59	75	70.2	64.7
MPV (femtoliters)	>11.5	29	96	87.9	57.5

## Discussion

CAD is a leading cause of morbidity and mortality worldwide. Platelets play a crucial role in the development and progression of CAD [9]. Abnormal platelet function is a major factor contributing to coronary events [10]. An increase in platelet size is associated with heightened susceptibility to reinfarctions [11]. Hematology analyzers are commonly used to determine MPV, a key platelet parameter that can effectively predict CAD [12]. Studies have shown that platelet parameters, particularly MPV and PDW, are significantly elevated in patients with ACS compared to controls, making them easily accessible and affordable laboratory tests [13.] Larger platelets, which exhibit heightened hemostatic activity, are a risk factor for coronary thrombosis and subsequent myocardial infarction. These larger platelets can be identified through platelet volume indices (PVIs) [14]. Despite the elevated MPV and PDW levels in patients with ACS, the degree of CAD - determined by echocardiography, ECG abnormalities, and coronary angiography - does not correlate with these elevated parameters. Thus, in CAD patients, these measures should not be considered risk factors for CAD [15]. The hemostatic activity of larger platelets increases the risk of coronary thrombosis, which can lead to myocardial infarction. In ischemic heart disease, platelet count and PVIs can serve as markers for prethrombotic conditions [16]. Platelet indices, particularly MPV and PDW, are found to be higher in individuals who have suffered ST-segment elevation myocardial infarction and non-ST-segment elevation myocardial infarction compared to patients with unstable angina [17]. These PVIs are useful in indicating prethrombotic conditions in CAD [18]. Larger platelets can be easily detected during routine hematological tests and may benefit from preventive medication. PVI is a simple, cost-effective approach for predicting impending acute episodes [19]. Elevated platelet counts and indices are associated with adverse CAD outcomes.

In our study, MPV, PDW, and PLCR were significantly higher in CAD patients compared to controls, correlating well with similar studies. Platelets play an essential role in thrombosis development in CAD. Our findings showed higher platelet count, lymphocyte count, and PLR in CAD patients compared to controls [6].

The altered morphology and function of platelets are linked to the pathogenesis of CAD. Upon contact with ruptured plaques, platelets become larger and more active. These larger platelets produce more thromboxane A2, a potent promoter of thrombus formation, and are more metabolically and enzymatically active than smaller platelets [20]. Platelet activation results in conformational changes in GP IIb/IIIa receptors, which enhance fibrinogen binding. Fibrinogen, a multivalent molecule, facilitates platelet aggregation by bridging two platelets simultaneously. Consequently, larger platelets are released from the bone marrow due to increased platelet consumption at the site of the atherosclerotic plaque [20].

In our study, MPV showed a significant difference between CAD patients and the control group, with a mean ± SD of 10.75 ± 1.98 and 9.97 ± 1.00, respectively (p = 0.001, Table 1), consistent with studies by Reddy et al. [6], Khandekar et al. [14], Gururajaprasad et al. [20], Rodrigues et al. [21], and Sharma et al. [22] (9.37 ± 0.99 and 9.19 ± 0.62, respectively, with p < 0.001).

Additionally, we observed that an MPV cutoff value of ≥11.5 fL had 29% sensitivity and 96% specificity in predicting CAD, making it an important risk and prognostic factor (Table 3), similar to the findings by Reddy et al. [6].

PDW also showed a significant difference between the CAD and control groups with p = 0.002, consistent with studies by Reddy et al. [6], Celik et al. [11], Khandekar et al. [14], Rodrigues et al. [21], Gururajaprasad et al. [20], and Sharma et al. [22] (p < 0.005). PDW has been found to be a prognostic marker for evaluating mortality in patients following acute myocardial infarction [11]. In our study, the PDW cutoff value of <8.3 showed 45% sensitivity and 93% specificity, similar to the study by Reddy et al. [6].

PLCR also showed a significant difference between the CAD and control groups with p < 0.001, similar to studies by Reddy et al. [6], Khandekar et al. [14], Rodrigues et al. [21], Gururajaprasad et al. [20], and Ranjith et al. [23] (p < 0.001). PLCR, characterized by platelets >12 fL, has been found to be a useful marker in predicting CAD. In our study, a PLCR cutoff value of >29 showed 59% sensitivity and 75% specificity, similar to findings by Reddy et al. [6].

Studies by Reddy et al. [6], Khandekar et al. [14], Gururajaprasad et al. [20], and Rodrigues et al. [21] indicate that higher MPV, PDW, and PLCR values are useful markers for predicting CAD. These parameters are simple, economical, and have no contraindications, which aligns with our study findings.

Limitations

This study was conducted at a single institution with a small sample size, and data collection spanned only six months. Confounding factors such as hypertension, diabetes, family history, and dyslipidemia were not considered. A larger, multicenter study is recommended to validate these findings and enhance their generalizability.

## Conclusions

CAD can be predicted using easily available PVIs (MPV, PDW, and PLCR). Further studies should explore the integration of platelet indices with traditional cardiac biomarkers to improve early CAD risk assessment. Patients with larger platelets can be readily identified during routine hematological examinations and may benefit from preventive treatment. PVI represents an important, simple, and cost-effective tool for predicting acute cardiac events.
